# Concomitant overexpression of triple antioxidant enzymes selectively increases circulating endothelial progenitor cells in mice with limb ischaemia

**DOI:** 10.1111/jcmm.14287

**Published:** 2019-04-11

**Authors:** Lingjuan Liu, Yuqi Cui, Xin Li, Xingyi Que, Yuan Xiao, Chunlin Yang, Jia Zhang, Xiaoyun Xie, Peter J. Cowan, Jie Tian, Hong Hao, Zhenguo Liu

**Affiliations:** ^1^ Department of Cardiology Children's hospital of Chongqing Medical University Chongqing China; ^2^ Center for Precision Medicine and Division of Cardiovascular Medicine University of Missouri School of Medicine Columbia Missouri; ^3^ Department of Surgery University of Missouri School of Medicine Columbia Missouri; ^4^ Department of Medicine University of Melbourne Melbourne Australia; ^5^ Immunology Research Centre St. Vincent’s Hospital Melbourne Australia

**Keywords:** angiogenesis, EPCs, Gpx‐1, limb ischaemia, ROS, SOD1, SOD3

## Abstract

Endothelial progenitor cells (EPCs) are a group of heterogeneous cells in bone marrow (BM) and blood. Ischaemia increases reactive oxygen species (ROS) production that regulates EPC number and function. The present study was conducted to determine if ischaemia‐induced ROS differentially regulated individual EPC subpopulations using a mouse model concomitantly overexpressing superoxide dismutase (SOD)1, SOD3 and glutathione peroxidase. Limb ischaemia was induced by femoral artery ligation in male transgenic mice with their wild‐type littermate as control. BM and blood cells were collected for EPCs analysis and mononuclear cell intracellular ROS production, apoptosis and proliferation at baseline, day 3 and day 21 after ischaemia. Cells positive for c‐Kit^+^/CD31^+^ or Sca‐1^+^/Flk‐1^+^ or CD34^+^/CD133^+^ or CD34^+^/Flk‐1^+^ were identified as EPCs. ischaemia significantly increased ROS production and cell apoptosis and decreased proliferation of circulating and BM mononuclear cells and increased BM and circulating EPCs levels. Overexpression of triple antioxidant enzymes effectively prevented ischaemia‐induced ROS production with significantly decreased cell apoptosis and preserved proliferation and significantly increased circulating EPCs level without significant changes in BM EPC populations, associated with enhanced recovery of blood flow and function of the ischemic limb. These data suggested that ischaemia‐induced ROS was differentially involved in the regulation of circulating EPC population.

## 1 INTRODUCTION

Formation of new blood vessels (neovascularization) is an important mechanism in response to ischemic injuries/conditions such as ischemic heart disease and peripheral artery disease.^1^, ^2^ Bone marrow (BM)‐derived endothelial progenitor cells (EPCs) play a critical role in vascular re‐endothelialization, angiogenesis and prevention of neointima formation after vascular injury.^1^, ^2^ However, EPCs are a group of very heterogeneous cell population with a variety of different cell markers reported in the literature.^3^ There are also multiple sources for EPCs with BM and blood as the two major EPC sources.^4^

Reactive oxygen species (ROS) such as superoxide anion (O_2_^‐^) and hydrogen peroxide (H_2_O_2_) are critically involved in cell growth, migration, differentiation, apoptosis and senescence.^5^, ^6^ It has been reported that increased amounts of ROS is produced in response to tissue ischaemia.^8^ Although high concentrations of ROS are involved in senescence and apoptosis of endothelial cells and stem/progenitor cells and associated with defective neovascularization,^9^, ^10^ low levels of ROS generated during tissue ischaemia serve as intracellular signals to trigger angiogenesis.^11^, ^12^ The effects of ROS following limb ischaemia on EPCs are reported to be mainly through Nox2‐containing NADPH oxidase.^10^, ^13^, ^14^ However, the changes in specific subpopulations of EPCs in response to ROS formation during limb ischaemia have not been studied. It has been reported that ROS production in BM derived mononuclear cells is associated with the number of EPC.^14^ Many antioxidant enzymes including copper–zine superoxide dismutase (SOD1) in the cytoplasm,^16^ extracellular SOD (SOD3)^17^, ^18^ and glutathione peroxidase (Gpx‐1) 19, 20 have been individually reported to have a beneficial effect on the mobilization of EPCs and neovascularization after limb ischaemia. However, it has been reported that SOD overexpression could be associated with increased levels of hydrogen peroxide (H_2_O_2_) with increased oxidative stress in vitro and in vivo.^21^ Thus, it is not clear if the beneficial effect of SOD overexpression on EPC and neovascularization was indeed because of decreased ROS formation. It is known that SOD1, SOD3 and Gpx‐1 work together as an antioxidant network (AON) to eliminate ROS. Briefly, O_2_^‐ ^is readily dismutated by SOD into H_2_O_2_, which could then be converted into hydroxyl radicals by a Fenton‐type reaction or scavenged by either catalase or Gpx‐1.^22^ The combined effect of these antioxidant enzymes on individual EPC subpopulations following limb ischaemia has not been studied.

In the present study, experiments were conducted to test the hypothesis that ischaemia‐induced ROS could differentially regulate individual EPC subpopulations. As SOD overexpression could paradoxically increase the level of H_2_O_2_ with increased oxidative stress in vitro and in vivo,^21^ a triple‐transgenic mouse model (TG) with global concomitant overexpression of SOD1, SOD3 and Gpx‐1 with decreased ROS production was used to avoid potential increase of H_2_O_2_.^23^ Our previous study showed that concomitant overexpression of SOD1, SOD3 and Gpx‐1 had no measurable effect on the basal ROS level and baseline EPC populations in either BM or circulation.^23^ As the basal levels of ROS and EPC populations were low in mice, limb ischaemia was induced by femoral artery ligation (FAL) to increase ROS production and EPC levels. Although a variety of pathological conditions such as hyperlipidaemia, diabetes mellitus and infection could increase ROS generation,^24^, ^25^ we used the limb ischaemia model in consideration of the important role of EPCs in angiogenesis and vascular repair after ischemic injuries. As there were no unified cell markers for EPCs, we used four different groups of cell markers to identify EPCs to ensure a comprehensive analysis of EPCs in BM and blood.

## 2 MATERIALS AND METHODS

### 2.1 Animal models

All the animal experiments were performed in accordance with the ‘Guide for the Care and Use of Laboratory Animals of the US National Institutes of Health’. The experimental protocols for the present study were reviewed and approved by the Institutional Animal Care and Use Committee of the University of Missouri School of Medicine, Columbia, MO, USA. To evaluate the role of ROS formation in the changes in the populations of EPCs in response to limb ischaemia, a triple‐transgenic (TG) mouse model (with C57 BL/6 background) with concomitant global overexpression of an AON composed of SOD1, SOD3 and Gpx‐1 (6‐8 weeks old, male) with decreased ROS production was used in the present study, with the littermate wild‐type (WT) male C57BL6 mice as the control as detailed previously.^27^ Successful creation of the TG mice was confirmed with genotyping (Figure S1A) and increased protein expression of the enzymes (using western blot analysis) (Figure S1B) as well as increased enzyme activities as described.^22^, ^23^ Of note, the antibodies for SOD‐1 (Invitrogen, Catalogue: MA1‐105), SOD‐3 (Santa Cruz, Catalogue: sc‐376948) and Gpx‐1 (Invitrogen, Catalogue: 702762) used for the present study were cross‐reactive with the respective proteins from both human and mouse. Thus, the basal expression levels for these three enzymes were detectable in the wild‐type littermate (Figure S1B).

### 2.2 Hind limb ischaemia and blood flow measurement

FAL was performed to produce hind limb ischaemia in the mice as described.^28^ Mice were anaesthetized with one dose of Ketamine 100 mg/kg (0.1 mg/g, ip) mixed with Xylazine 20 mg/kg (0.02 mg/g, ip). 1.25% isoflurane/O_2_ was inhaled to induce anaesthesia. The hind limbs were depilated. The animal's body temperature was maintained at 37 ± 0.5°C. The left femoral artery was exposed through a 2‐mm incision without retraction and with minimal tissue disturbance. A 7‐0 ligature was placed distal to the origin of the lateral caudal femoral and superficial epigastric arteries (the latter was also ligated) and proximal to the genu artery. The femoral artery was transected between the sutures and separated by 1‐2 mm. The wound was irrigated with saline and closed. Laser Doppler perfusion imaging (LDPI, Moor Instruments, Devon, UK) was used to determine the total local blood perfusion in the limbs pre‐operatively, immediately post‐operatively and at day 3, 7, 14 and 21 post‐operatively. Excessive hair was removed from the limbs before imaging and the mice were placed on a heating pad at 37°C to minimize temperature variation. Right limb blood flow was also measured as control. The ratio of blood flow (left ischemic limb blood flow/right normal limb blood flow) was used to monitor the blood flow recovery.

### 2.3 Treadmill performance

Wild‐type C57BL/6J and TG male mice were subjected to a treadmill running test following a protocol modified from that of Massett and Berk.^29^ Mice were placed on a rodent treadmill equipped with an electric grid at the rear and were made to run continuously until exhaustion at day 7, 14 and 21 after limb ischaemia (indicated by falling on the electric grid twice) and the running time on the treadmill was recorded. Two separate trials with eight WT and eight TG mice per trial were conducted. Both TG and WT mice were able to run more than 400 minutes at baseline before limb ischaemia.

### 2.4 ***CD31*** ***immunohistochemistry and assessment of capillary density***

For CD31 immunohistochemistry analysis, at day 3, 7 and 21 after left hind limb ischaemia, limb muscle tissues were fixed with 4% paraformaldehyde in PBS and sectioned with 5 μm thickness and incubated overnight with rat anti‐mouse biotinylated endothelial marker CD31 (1/200, BD Biosciences) followed by a biotin‐conjugated secondary antibody (goat anti‐rat, dilution 1:300; AbCam). The reaction was enhanced with tyramine amplification and the avidin–biotin–horseradish–peroxidase system and visualized using NovaRED. Sections of mouse hind limb before ligation were used as control. The number of cells positive for each cell marker was counted on at least four pictures per mouse. Each section was viewed with the pictures taken both at 20× under microscope. For vascular density quantification (capillaries and collaterals), non‐overlapping 5‐8 fields were captured as described.^30^, ^31^ Capillaries were counted using the Image J software.

### 2.5 *Intracellular ROS* *detection*

BM and blood cells were harvested from both WT and TG mice at day 3 and 21 after limb ischaemia. Healthy and age‐matched mice without limb ischaemia were used as control. Red blood cells (RBC) were eliminated using RBC lysis as described.^33^ The level of intracellular ROS formation in blood cells after limb ischaemia was determined using the ROS Detection Reagents‐FITC (Invitrogen) as described.^34^ The cells were incubated with the reagent for 10 minutes at 37°C. The labelled cells were washed twice with PBS and then suspended in warm PBS for analysis using flow cytometry. The fluorescence‐positive cells were quantitatively evaluated using an LSRII (BD Bioscience, CA) at a wavelength of 525 nm as described.^35^

### 2.6 *Analysis of mouse EPC* *populations and mononuclear cell apoptosis and proliferation*

To determine the effect of limb ischaemia on the population of EPCs, BM and blood cells were harvested in the mice at day 3 and 21 after limb ischaemia. Healthy and age‐matched mice without limb ischaemia served as control. After elimination of RBC with RBC lysis buffer, multicolour analysis for BM and circulating EPCs was performed using an LSRII system (BD Biosciences, CA). A variety of cell surface markers and their combinations for identification of BM and circulating EPCs were used as described,^36^, ^37^ including CD34^+^/Flk‐1^+^, Sca‐1^+^/Flk‐1^+^, c‐Kit^+^/CD31^+^ and CD34^+^/CD133^+^. The cell populations were carefully compensated (each cell population percentile was further confirmed with single antibody staining and fluorescence minus one with the isotype antibody as the control) and applied to all samples. The total cell population was gated and each EPC population with specific double‐positive markers was analysed using flow cytometry as described.^43^, ^44^ All antibodies were obtained from Biolegend (San Diego, CA) except Flk‐1 APC‐Cy7 from Becton Dickinson Biosciences (NJ) and CD34 FITC from eBioscience (San Diego, CA). The anti‐mouse lineage cocktail from Biolegend included anti‐mouse CD3, clone 17A2, anti‐mouse Ly‐6G/Ly‐6C, clone RB6‐8C5, anti‐mouse CD11b, clone M1/70, anti‐mouse CD45R/B220, clone RA3‐6B2, anti‐mouse TER‐119/Erythroid cells and clone Ter‐119.

To evaluate BM and circulating mononuclear cell apoptosis, murine BM and blood was collected at day 3, 7 and 21. After elimination of RBC with RBS lysis, blood cell apoptosis was determined with FACS using the apoptosis kit from BD Pharmingen (CA). The early apoptotic cells were defined as Annexin V FITC positive cells, whereas the late apoptotic cells were characterized as Annexin V FITC and propidium iodide (PI) double‐positive cells as described.^45^ For BM and blood mononuclear proliferation analysis, mice were injected (ip) with 1 mg BrdU 12 hours before analysis.^46^ Cells were permeabilized and stained with anti‐BrdU PE‐Cy5 using the BrdU Flow Kit as per manufacturer's instruction (559619, BD Biosciences, San Jose, CA). For mononuclear cell proliferation analysis, the BrdU positive BM and blood mononuclear cells were analysed using flow cytometry.^47^ For all FACS data, at least 5 × 10^4^ cells were counted for each sample with a total of at least 1 × 10^6^ cells per sample.

### 2.7 Statistical analysis

All the data were presented as means ± standard deviation (SD), two way ANOVA (analysis of variance) (PRISM Version 4.0.; GraphPad Software, Inc, San Diego, CA) followed by Bonferroni post‐tests was used for comparing the subgroups of data from TG and WT mice to minimize type I error as appropriate for all data. The differences were considered statistically significant when a two‐tailed *P* < 0.05.

## 3 RESULTS

### 3.1 Concomitant overexpression of triple antioxidant enzymes significantly enhanced the recovery of blood flow and function of the ischemic limb

**Figure 1:**
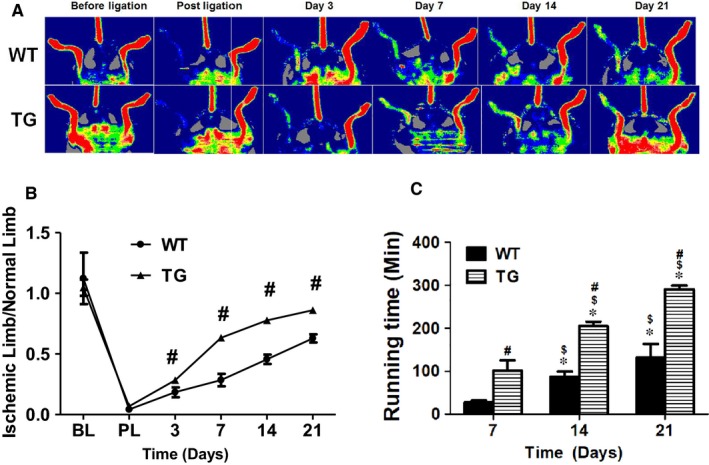
TG mice exhibited enhanced recovery of blood flow and function after limb ischaemia. Left hind limb femoral artery was transected to induce limb ischaemia in C57BL/6 and TG mice. Mouse hind limb blood flow was measured with LDPI before, immediately after ligation and at day 3, 7, 14 and 21 (A). The blood flow recovery was significantly increased in TG mice compared to the WT mice (B). After surgically induced hind limb ischaemia, C57BL/6 and TG mice were placed on treadmill to test the limb function at day 7, 14 and 21. The TG mice were able to run for a significantly longer time than WT mice (C). WT: C57BL/6 mice; TG: triple‐transgenic mouse with overexpression of antioxidant network composed of superoxide dismutase (SOD)1, SOD3 and glutathione peroxidase (Gpx‐1). Ischemic limb/normal limb: ratio of mouse ischemic left hind limb blood flow to normal right hind limb blood flow. BL: before ligation. PL: post ligation. # WT vs TG, *P* < 0.001, n = 8; *Day 14 or 21 vs day 7, *P* < 0.01, n = 8; $ Day 21 vs 14, *P* < 0.01, n = 8; #WT vs TG, *P* < 0.01, n = 8

The recovery of blood flow and muscle function in the mice following acute hind limb ischaemia was evaluated. There was no measurable blood flow in the ischemic limb immediately after femoral artery ligation as expected in both WT C57 BL/6 mice and TG mice with concomitant global overexpression of SOD1, SOD3 and GSHPx‐1 (Figure 1A), confirming successful creation of limb ischemic model. The blood flow was recovered gradually after ligation in WT mice with 18% and 62% of blood flow recovered at day 3 and day 7 post ischaemia respectively (Figure 1B). The function of the ischemic limb as determined by the running time on treadmill was gradually recovering after day 7 of ischaemia with the running time of 27 ± 14 min that increased to 131 ± 79 min at day 21 (much less than that of over 400 min for healthy and age‐matched control mice) (Figure 1C). A significantly enhanced recovery of blood flow (Figure 1A,B) and running time (Figure 1C) of the ischemic limb was observed in the TG mice with concomitant overexpression of SOD1, SOD3 and Gpx‐1. Indeed, the blood flow was significantly increased in TG mice within only 3 days after ischaemia and continued to raise significantly to 0.86 ± 0.01 at day 21 after ischaemia (0.63 ± 0.03 for WT mice) (Figure 1).

### 3.2 Concomitant overexpression of triple antioxidant enzymes effectively preserved the muscle fibres of the ischemic limb

**Figure 2:**
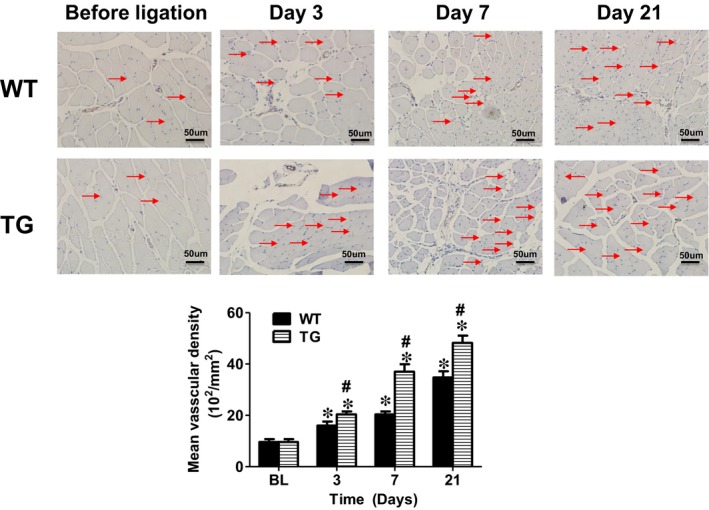
Concomitant overexpression of triple antioxidant enzymes effectively preserved the muscle fibres of the ischemic limb. The muscle fibres reduced in size and became largely disconnected in the WT control mice with limb ischaemia. CD31 staining showed that capillary density was low in the limb muscle after ischaemia for 3, 7 and 21 days. On the other hand, the muscle fibres appeared to remain long and large, as well as well‐connected in the limb muscles at day 21 after ischaemia in the TG mice with concomitant global overexpression of SOD1, SOD3 and Gpx‐1. CD31 staining demonstrated that the capillary density remained largely intact (similar to the non‐ischemic limb). Normal: normal right limb; ischaemia; ischemic left limb. Red arrow: capillary vessels. BL: before ligation. Scale bar: 50µm

As the induction of limb ischaemia leads to remarkable muscle degeneration,^48^ the limb muscle morphology was examined. The muscle fibres reduced in size and became largely disconnected in the WT control mice with limb ischaemia. On the other hand, the muscle fibres appeared to remain long and large, as well as connected nicely in the limb muscles at day 21 after ischaemia in the TG mice with concomitant global overexpression of SOD1, SOD3 and Gpx‐1 (Figure 2). CD31 staining showed that the capillary density in the ischemic muscle gradually increased from day 3 to day 21 in WT mice (Figure 2). Whereas in the TG mice, the muscle capillary density was significantly increased compared to the WT mice after limb ischaemia: 20.4 ± 1.1 (10^2^/mm^2^) vs 16 ± 1.6 (10^2^/mm^2^), 37 ± 2.9 (10^2^/mm^2^) vs 20.4 ± 1.1 (10^2^/mm^2^) and 48.2 ± 2.9 (10^2^/mm^2^) vs 34.8 ± 2.4 (10^2^/mm^2^) for day 3, 7 and 21 post ischaemia respectively (n = 8, *P* < 0.01). The capillary density appeared to be largely intact in the TG mice at day 21 after ischaemia (similar to the non‐ischemic limb) (Figure 2).

### 3.3 Concomitant overexpression of triple antioxidant enzymes effectively prevented intracellular ROS formation after limb ischaemia

**Figure 3:**
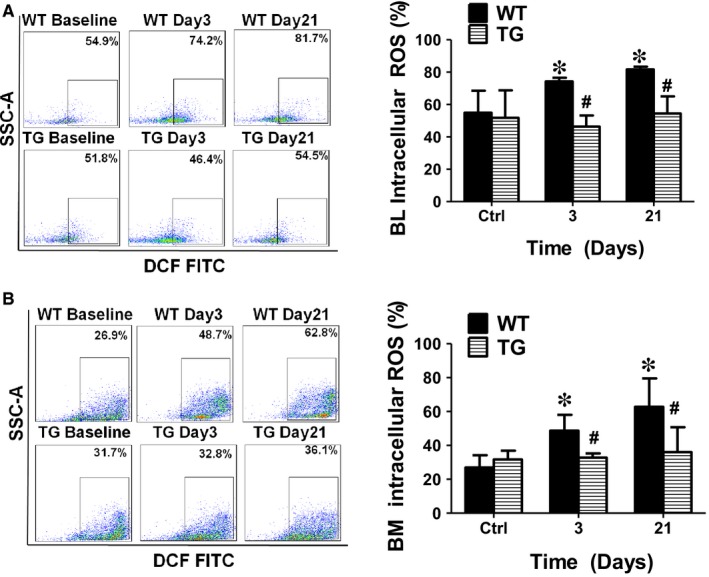
Concomitant overexpression of triple antioxidant enzymes effectively inhibited intracellular ROS generation after limb ischaemia. Both bone marrow (BM) and blood intracellular ROS were significantly increased in the WT control mice at day 3 to day 21 after limb ischaemia. The blood and BM intracellular ROS production after limb ischaemia was effectively blocked in the mice overexpressing the antioxidant enzymes. BL: blood. BM: bone marrow. *Day 3 or 21 vs Ctrl, *P* < 0.05, n = 8; $ Day 21 vs 3, *P* < 0.05, n = 8; #WT vs TG, *P* < 0.05, n = 8

It is known that limb ischaemia significantly increases ROS production^49^ and the ROS production in BM derived mononuclear cells is associated with the number of EPC.^14^ To reduce ischaemia‐induced ROS production, a TG mouse model overexpressing the antioxidant enzyme network (AON) with reduced ROS formation was used.^23^ As expected, both BM and blood mononuclear cell intracellular ROS were significantly increased in the WT control mice at day 3 and day 21 after limb ischaemia. Concomitant overexpression of triple antioxidant enzymes in the TG mice effectively blocked the intracellular ROS production in both blood and BM mononuclear cells after limb ischaemia (Figure 3A,B). Of note, significantly increased protein levels for SOD1, SOD3 and Gpx‐1 were observed in the BM cells on Western blot analysis as expected (Figure S1B), confirming that these three enzymes were indeed overexpressed in BM cells as well as in TG mice.

### 3.4 Concomitant overexpression of triple antioxidant enzymes selectively maintained circulating EPCs at elevated level without significant change in bone marrow EPC population after limb ischaemia.

**Figure 4:**
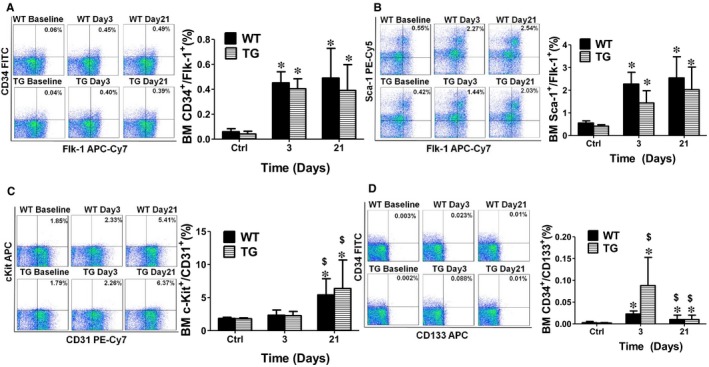
Concomitant overexpression of triple antioxidant enzymes did not significantly change the bone marrow EPCs population after limb ischaemia. Flow cytometry analysis showed that bone marrow EPCs (BM EPCs) level including CD34^+^/Flk‐1^+ ^and Sca‐1^+^/Flk‐1^+^ were significantly increased up to 7‐ and 4‐ fold at day 3 after ischaemia respectively, and maintained at the same level at day 21 in WT mice (A and B). Although there was no change in the population of BM c‐Kit^+^/CD31^+ ^cells at day 3 after limb ischaemia, this cell population was significantly increased at day 21 (C). The population of BM CD34^+^/CD133^+ ^cells slightly increased at day 3 after ischaemia (0.02 ± 0.01%) and then dropped at day 21 (0.02 ± 0.01%) (D). However, no significant changes were observed in the populations of BM EPCs including the cells positive for CD34^+^/Flk‐1^+^, Sca‐1^+^/Flk‐1^+ ^and c‐Kit^+^/CD31^+ ^in TG mice compared to the WT mice (A, B and C), except for CD34^+^/CD133^+ ^cells that were significantly increased at day 3 after ischaemia and then decreased to the same level as WT mice at day 21 (D). BL: blood. BM: bone marrow. *Day 3 or 21 vs Ctrl, *P* < 0.05, n = 8; $ Day 21 vs 3, *P* < 0.05, n = 8; #WT vs TG, *P* < 0.05, n = 8. *Day 3 or 21 vs Ctrl, *P* < 0.05, n = 8; $ Day 21 vs 3, *P* < 0.05, n = 8; #WT vs TG, *P* < 0.05, n = 8.

**Figure 5:**
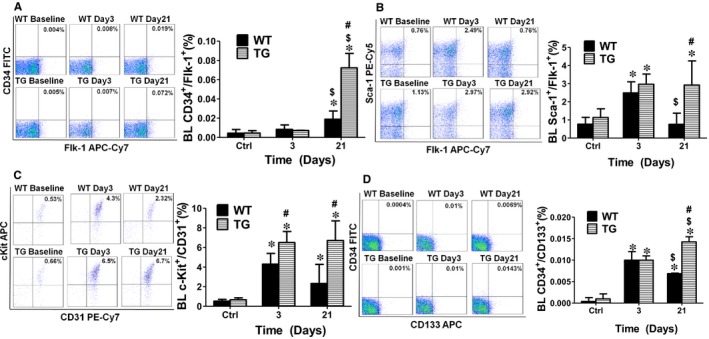
Concomitant overexpression of triple antioxidant enzymes helped maintain circulating EPCs at elevated levels after limb ischaemia. Flow cytometry analysis showed that the CD34^+^/Flk‐1^+^ cell population stayed at an extremely low level shortly after limb ischaemia and only slightly increased at day 21 (A). On the other hand, circulating Sca‐1^+^/Flk‐1^+ ^cells and CD34^+^/CD133^+ ^cells were initially increased at day 3, but significantly decreased at day 21 (B and D). When ischaemia‐induced ROS production was inhibited in TG mice, all the circulating EPCs populations in TG mice were significantly increased up to 3‐fold at day 21 after ischaemia over the WT mice. The blood c‐Kit^+^/CD31^+ ^cell population was elevated to 6.5 ± 1.13% in the TG mice (4.3 ± 1.1% in WT mice) at day 3 after ischaemia (C). TG mice maintained circulating EPCs at elevated level compared to the WT mice. BL: blood. BM: bone marrow. *Day 3 or 21 vs Ctrl, *P* < 0.05, n = 8; $ Day 21 vs 3, *P* < 0.05, n = 8; #WT vs TG, *P* < 0.05, n = 8

To evaluate the role of ROS in the regulation of individual EPC populations, both BM and blood cells were collected to analyse the EPC populations. Flow cytometry analysis showed that BM EPC levels including CD34^+^/Flk‐1^+ ^and Sca‐1^+^/Flk‐1^+^ were significantly increased up to 7‐ and 4‐fold at day 3 after ischaemia respectively, and stayed at the same levels up to day 21 in the WT mice (Figures 4 and 5). Although there was no change in the population of BM c‐Kit^+^/CD31^+ ^cells at day 3 after limb ischaemia, this cell population was significantly increased at day 21 (Figure 4C). The population of BM CD34^+^/CD133^+ ^cells slightly increased at day 3 after ischaemia (0.02 ± 0.01%) and then dropped at day 21 (0.02 ± 0.01%) (Figure 4D). Very interestingly, the populations of circulating Sca‐1^+^/Flk‐1^+ ^cells and CD34^+^/CD133^+ ^cells initially increased at day 3, but significantly decreased at day 21 (Figure 4B,D). On the other hand, the CD34^+^/Flk‐1^+^ cell population stayed at an extremely low level shortly after limb ischaemia and only slightly increased at day 21 (Figure 4A).

All circulating EPC populations in TG mice were significantly increased up to 3 folds at day 21 over the WT mice following hind limb ischaemia (Figure 5). And the ischaemia‐induced ROS production was also inhibited by TG mice (Figure 3). The blood c‐Kit^+^/CD31^+ ^cell population was elevated to 6.5 ± 1.13% in the TG mice (4.3 ± 1.1% in WT mice) at day 3 after ischaemia (Figure 5C). However, there were no significant differences in the populations of BM EPCs including CD34^+^/Flk‐1^+^, Sca‐1^+^/Flk‐1^+ ^and c‐Kit^+^/CD31^+ ^in TG mice compared to the WT mice (Figure 4). However, CD34^+^/CD133^+ ^was significantly increased at day 3 after ischaemia and then decreased to the same level as WT mice at day 21 (Figure 4D).

### 3.5 Concomitant overexpression of triple antioxidant enzymes inhibited apoptosis and promoted proliferation of bone marrow and circulating mononuclear cells

**Figure 6:**
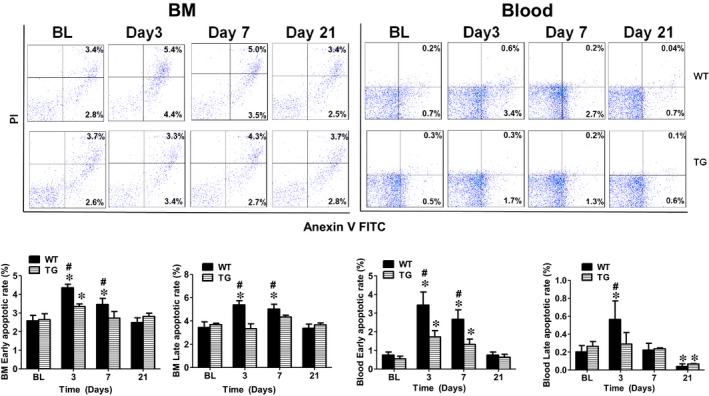
Concomitant overexpression of triple antioxidant enzymes prevented BM and circulating mononuclear cell apoptosis after limb ischaemia. Flow cytometry analysis showed that BM and circulating early and late mononuclear cell apoptotic rates were significantly increased in WT mice at day 3 and day 7 compared with TG mice. BM: bone marrow. *Day 3, 7 or 21 vs BL, *P* < 0.05, n = 8; #TG vs WT, *P* < 0.05, n = 8; BL: before ligation

**Figure 7:**
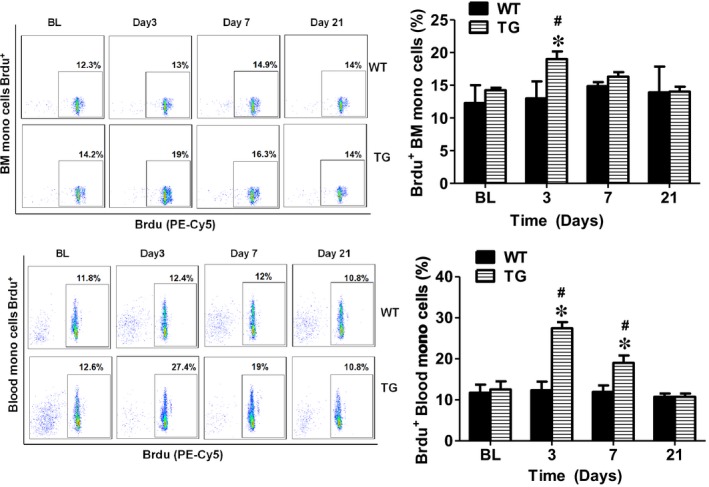
Concomitant overexpression of triple antioxidant enzymes promoted BM and circulating mononuclear cell proliferation after limb ischaemia. Flow cytometry analysis showed that BM and circulating mononuclear cell proliferation rate were significantly increased in TG mice at day 3 and day 7 compared with WT mice. *Day3, 7 or 21 vs BL, *P* < 0.05, n = 8; #TG vs WT, *P* < 0.05, n = 8; BL: before ligation

To further explore the mechanisms on how AON in the TG mice maintained the circulating EPCs at a higher level compared to the WT mice, BM and blood cells were collected at day 3, 7 and 21 following limb ischaemia to analyse the mononuclear cell apoptotic and proliferation rate. As shown in Figure 6, in the BM, both early (4.4 ± 0.2%) and late (5.4 ± 0.4%) mononuclear call apoptotic rate were significantly increased at day 3 after limb ischaemia and remained constant level at day 7 (3.5 ± 0.3% and 5.0 ± 0.4%) and then decreased to the same level as control. However, there were no changes in the BM mononuclear apoptotic rate in TG mice for 21 days after limb ischaemia except for a slight increase in the early apoptotic rate at day 3 (Figure 6). Similar results were observed for the blood circulating mononuclear cells with their early apoptotic rate significantly elevated at day 3 and 7, whereas late apopotitc rate increased at day 3 in WT mice. Both early and late apoptotic rates for the circulating mononuclear cells in TG mice were significantly lower compared to the WT mice (Figure 6). On the other hand, the cell proliferation rate for BM (TG 19 ± 1.2% vs WT 13 ± 2.6%) and circulating mononuclear cells (TG 27.4 ± 1.5% vs WT 12.4 ± 2.1%) was significantly increased in TG mice at day 3 compared with WT mice (Figure 7). The cell proliferation rate for circulating mononuclear cells in TG mice stayed elevated compared with the WT mice up to day 7 (19 ± 1.8% vs 12 ± 1.6%).

## 4 DISSCUSSION

In the present study, we demonstrated that limb ischaemia increased ROS production in both circulating and BM mononuclear cells and significantly increased EPCs levels in BM and blood as expected. Concomitant overexpression of triple antioxidant enzymes (SOD1, SOD3 and Gpx‐1) effectively blocked ischaemia‐induced mononuclear cell ROS production, apoptosis and promoted proliferation of circulating and BM mononuclear cells. After limb ischaemia, the circulating EPCs level was kept at significantly elevated level for up to 21 days in the mice with concomitant overexpression of triple antioxidant enzymes, whereas the circulating EPC in WT mice initially increased at day 3 and then decreased after limb ischaemia, although the change of BM EPC in TG mice was similar to that in WT mice. The mice with concomitant overexpression of triple antioxidant enzymes also exhibited a significantly enhanced recovery of blood flow and function of the ischemic limb compared to the WT mice. This was the first time to report that ischaemia‐induced ROS was preferentially involved in the regulation of circulating EPCs, not BM EPCs, using a mouse model with concomitant overexpression of SOD1, SOD3 and Gpx‐1 and reduced ROS formation.

Angiogenesis following limb ischaemia is an important mechanism for limb recovery.^1^, ^2^ BM‐derived circulating EPCs are capable of differentiating into endothelial cells 50 and contributing to vasculogenesis with improved limb circulation.^51^ Similar effects were observed for human cord blood‐derived EPCs in nude mouse model with limb ischaemia.^52^ ROS such as O_2_^‐ ^and H_2_O_2_ play an important role in normal cell growth, migration, differentiation, apoptosis and senescence.^7^ ROS at low levels functions as signalling molecules and potentially promotes angiogenesis and vasculogenesis.^13^, ^24^, ^25^ Our data showed that after limb ischaemia, both BM and circulating EPC levels were significantly increased along with a significant increase in ROS production as expected.^49^ Excess amounts of ROS are toxic and involved in endothelial and stem/progenitor cell senescence and apoptosis.^26^ It has been shown that the number and function of EPCs are reduced in a variety of cardiovascular diseases, including hypertension, atherosclerosis, diabetes, coronary artery disease and heart failure, as well as comorbid risk factors such as ageing, hypercholesterolemia, cigarette smoking and air pollution,^53^, ^54^ all of which are associated with oxidative stress. However, the changes in EPC populations in response to oxidative stress in different conditions varied significantly. We observed that treatment with oxidized low‐density lipoproteins (ox‐LDL) or hyperlipidaemia significantly increased ROS production and resulted in a ROS‐dependent increase in the circulating CD34^+^/Flk‐1^+^ cell population in mice.^23^ On the other hand, fine particulate matter (PM_2.5_) exposure could lead to a significant increase in ROS production and significant decrease in the population of circulating CD34^+^/CD133^+^ cells that was effectively prevented when ROS formation was inhibited in mice.^56^ In the present study, we observed a significantly increased ROS production after limb ischaemia that persisted for up to 21 days. The BM CD34^+^/CD133^+^, blood CD34^+^/CD133^+^ and Sca‐1^+^/Flk‐1^+^ cell populations were initially increased at day 3 after limb ischaemia, then returned to baseline level by day 21 with slow recovery of blood flow and limb function in WT mice. The reason for such a big difference in the role of ROS in the regulation of EPC populations at different settings is unclear. It is certainly possible that different species and/or amount of ROS are generated in response to different oxidative stress inducer, thus leading to various changes in EPCs populations. Further studies are needed to address this complex situation.

Three antioxidant enzymes SOD1, SOD3 and Gpx‐1 were reported to attenuate oxidative stress and might be essential for SCs and EPCs function and reparative neovascularization after ischaemia.^10^, ^16^, ^17^ However, SOD1, SOD3 and Gpx‐1 function as a team to reduce ROS production and minimize oxidative stress. Briefly, O_2_^‐^ is readily dismutated by SOD into H_2_O_2_, which can then be converted into hydroxyl radicals by a Fenton‐type reaction or scavenged by either catalase or Gpx‐1.^22^ However, there is a major concern that SOD overexpression itself could increase the level of hydrogen peroxide (H_2_O_2_) with increased oxidative stress in vitro and in vivo,^21^ making it difficult to interpret the data with SOD overexpression alone model. The present study showed that concomitant overexpression of SOD1, SOD3 and Gpx‐1 in TG mice effectively prevented ROS production in circulating and BM mononuclear cells in the setting of limb ischaemia and maintained BMSCs and circulating EPCs at elevated level associated with enhanced recovery of the ischemic limb with improved angiogenesis, blood flow and limb function. Similarly, many antioxidant reagents including 3‐methyl‐1‐phenyl‐2‐pyrazolin‐5‐1(MCI‐186),^57^ nifedipine,^58^ probucol 59 were also reported to promote vascular formation and reduce tissue ischaemia. However, one study showed that infusion of the antioxidant ebselen into WT mice significantly blocked the increase in blood flow and capillary density after ischaemia.^13^ Clearly, more studies are needed to address this inconsistent observation in the future.

It is known that certain level of ROS could be proangiogenic. However, excessive levels of ROS lead to vascular disease through direct and irreversible oxidative damage to macromolecules and disruption of redox‐dependent vascular wall signalling processes.^60^ It is also known that angiogenesis is a complex process and involves a variety of local and systematic factors and ischaemia could increase ROS levels both locally and systematically.^60^, ^61^ It is certainly possible that ischaemia‐induced ROS production in the local ischemic tissue and at the systematic level plays a different role in EPC population and/or function (such as mobilization, proliferation, migration and apoptosis, as well as homing). Other proangiogenic factors (both local and systematic) could also contribute to the improved angiogenesis and/or muscle regeneration in the mice (TG mice) with global expression of SOD1, SOD3 and Gpx1 in addition to the increased number of circulating EPCs. In fact, we observed that the levels of Akt1 and ERK1/2 phosphorylation were indeed significantly increased in the muscle in the TG mice with limb ischaemia as compared with wild‐type mice (unpublished observation). Although numerous studies have shown that circulating EPCs are able to improve the hind limb angiogenesis and ischemic muscular recovery,^51^, ^52^, ^60^, ^61^ further studies are needed to determine if and how circulating EPCs are directly involved in the increase in neovascularization in ischemic tissue and improved blood flow recovery in the TG mice. Further investigations are also needed to elucidate whether the improved angiogenesis in TG mice was secondary to other ischaemia‐associated proangiogenic molecules in this triple‐transgenic mouse model.

One of the interesting findings in the present study was that circulating EPCs benefited more from the protective effect of concomitant overexpression of SOD1, SOD3 and Gpx‐1 in TG mice than the bone marrow EPCs. The reason for the differential benefit for circulating EPCs in TG mice is unclear at this point. Of course, there are lots of other questions that need to be addressed as well especially the underlying mechanisms for the protective action of concomitant overexpression of the triple antioxidant enzymes on EPCs in TG mice. It is also critical to identify the specific SCs and EPCs population that are responsible for ischemic limb recovery. Further studies are needed to investigate other potential mechanisms involved in angiogenesis and limb ischaemia recovery in TG mice and to confirm our findings from the animal models with clinical study. Just like any transgenic animal models, one of the major concerns for the triple‐transgenic mouse model is the triggering of unknown compensatory mechanisms that may contribute to the changes in EPC populations. Thus, further studies are needed to evaluate the potential roles of the compensatory mechanisms in ROS formation, EPC populations and angiogenesis.

In conclusion, our data demonstrated that concomitant overexpression of SOD1, SOD3 and Gpx‐1 significantly attenuated ischaemia‐induced ROS production and selectively maintained an elevated level of the circulating EPCs, not the bone marrow EPCs, associated with enhanced recovery of blood flow and function of the ischemic limb. Further studies are needed to investigate the mechanisms for the selective beneficial effects of ROS reduction on circulating EPCs.

## CONFLICTS OF INTEREST

None.

## AUTHOR CONTRIBUTIONS

Lingjuan Liu measured the mouse hind limb blood flow and mouse running time and helped with data analysis; Yuqi Cui did the femoral artery ligation, FACS, immunohistochemistry, helped with data analysis and wrote the draft manuscript; Xin Li, Yuan Xiao, Jia Zhang, and Xiaoyun Xie prepared the FACS sample; Chunlin Yang did the mouse genotypying and Western blot; Dr Peter J. Cowan contributed the TG mouse line; Drs. Xingyi Que, Jie Tian and Hong Hao were involved in the experiment design and data analysis; and Dr Zhenguo Liu was responsible for the idea, involved in the experiment design, data analysis and interpretation and modification and revisions of the manuscript.
