# Supplementary figures and images for: Concomitant overexpression of triple antioxidant enzymes selectively increases circulating endothelial progenitor cells in mice with limb ischaemia
Source: J Cell Mol Med. 2019 Apr 11;23(6):4019–29. doi: 10.1111/jcmm.14287 (PMC6533526; doi:10.1111/jcmm.14287)

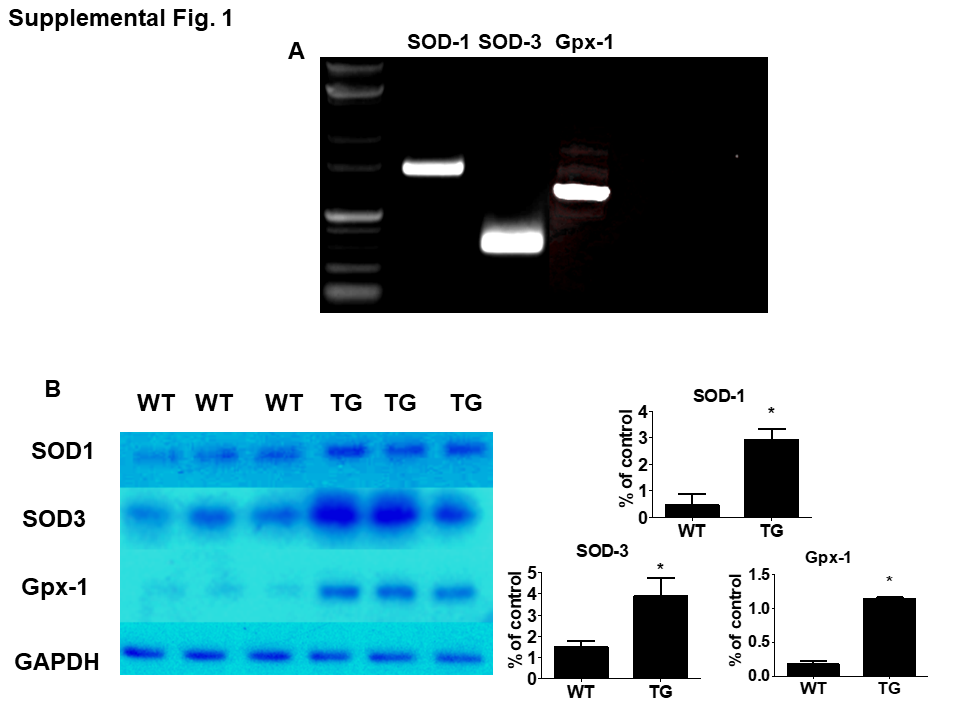

Supplement: Supplementary file 1 [file JCMM-23-4019-s001.tif]
